# Interpretation of course conceptual structure and student self-efficacy: an integrated strategy of knowledge graphs with item response modeling

**DOI:** 10.1186/s12909-024-05401-6

**Published:** 2024-05-23

**Authors:** Zhen-Yu Cao, Feng Lin, Chun Feng

**Affiliations:** 1 Department of Rehabilitation Medicine, School of Acupuncture-Moxibustion and Tuina, School of Health Preservation and Rehabilitation, Nanjing University of Chinese Medicine, 210023 Nanjing, China; 2 School of Rehabilitation Medicine, Nanjing Medical University, 211100 Nanjing, China; 3 School of Medicine, Tongji University, 200331 Shanghai, China; 4 The Center of Rehabilitation Therapy, The First Rehabilitation Hospital of Shanghai, Rehabilitation Hospital Affiliated to Tongji University, 200090 Shanghai, China

**Keywords:** Knowledge points (KPs), Item Response Theory (IRT), Knowledge graphs (KG), Teaching evaluation, Physical therapy

## Abstract

**Background:**

There is a scarcity of studies that quantitatively assess the difficulty and importance of knowledge points (KPs) depending on students’ self-efficacy for learning (SEL). This study aims to validate the practical application of psychological measurement tools in physical therapy education by analyzing student SEL and course conceptual structure.

**Methods:**

From the “Therapeutic Exercise” course curriculum, we extracted 100 KPs and administered a difficulty rating questionnaire to 218 students post-final exam. The pipeline of the non-parametric Item Response Theory (IRT) and parametric IRT modeling was employed to estimate student SEL and describe the hierarchy of KPs in terms of item difficulty. Additionally, Gaussian Graphical Models with Non-Convex Penalties were deployed to create a Knowledge Graph (KG) and identify the main components. A visual analytics approach was then proposed to understand the correlation and difficulty level of KPs.

**Results:**

We identified 50 KPs to create the Mokken scale, which exhibited high reliability (Cronbach’s alpha = 0.9675) with no gender bias at the overall or at each item level (*p > 0.05*). The three-parameter logistic model (3PLM) demonstrated good fitness with questionnaire data, whose Root Mean Square Error Approximation was < 0.05. Also, item-model fitness unveiled good fitness, as indicated by each item with non-significant *p-values* for chi-square tests. The Wright map revealed item difficulty relative to SEL levels. SEL estimated by the 3PLM correlated significantly with the high-ability range of average Grade-Point Average (*p < 0.05*). The KG backbone structure consisted of 58 KPs, with 29 KPs overlapping with the Mokken scale. Visual analysis of the KG backbone structure revealed that the difficulty level of KPs in the IRT could not replace their position parameters in the KG.

**Conclusion:**

The IRT and KG methods utilized in this study offer distinct perspectives for visualizing hierarchical relationships and correlations among the KPs. Based on real-world teaching empirical data, this study helps to provide a research foundation for updating course contents and customizing learning objectives.

**Trial registration:**

Not applicable.

**Supplementary Information:**

The online version contains supplementary material available at 10.1186/s12909-024-05401-6.

## Introduction

Knowledge points (KPs) serve as fundamental units within the realm of learning content, encompassing theories, ideas, thoughts, etc [1]. Determining the importance and difficulty of KPs is crucial for effective curriculum development [2]. Experts typically identify key KPs and peripheral KPs aligned with learning objectives [3]. Key KPs, or important points, are the core concepts of course content. Additionally, KPs can be classified as either complex or simple, considering their respective levels of teaching difficulty. Complex KPs, or difficult points, are challenging for students to master and require more education time [4]. Apart from teaching proficiency, KPs are considered a relative concept contingent upon student abilities [5]. Students with advanced abilities may identify certain KPs as relatively easy, while those with weaker abilities might find them comparatively challenging [6]. As a psychological attribute, the student’s ability is considered a “latent trait”, and is generally an inherent and intricate individual characteristic that cannot be directly measured by instruments or equipment.

In the context of learning theory, latent traits can usually be divided into two types, namely learning capacity and self-efficacy. Learning capacity specifies the capacity that one will produce positive learning outcomes, which can be manifested by the Grade-Point Average (GPA) [7]. Self-efficacy measured by psychometric questionnaires like the Learning Self-Efficacy Scale for Clinical Skills (L-SES) [8], reflects the belief in one’s ability to learn effectively [9].

Recent research has underscored the connection between high self-efficacy for learning (SEL) and successful academic performance [9–12]. However, there was still a knowledge gap regarding the varying degrees of difficulty that each student may experience when dealing with specific KPs. The existing tools, like the L-SES with its 12-item scale [8], primarily assess SEL but do not concurrently measure the difficulty of KPs. This limitation hinders the understanding of students’ learning experiences, as it overlooks the varying degrees of difficulty associated with specific KPs.

To address this gap, this study applied the Item Response Theory (IRT), a theoretical framework for considering person ability, and item difficulty on the same scale (in units of logit). The corresponding test analysis method is item response modeling (IRM), which can quantify how individuals with different levels of the latent trait are likely to respond to specific items. IRT can be broadly categorized into two main types, namely, non-parametric IRT (npIRT) and parametric IRT (pIRT) [13]. Compared to pIRT with explicit assumptions, the npIRT is more flexible in handling data and makes fewer assumptions about the underlying structure of the item responses. The npIRT may focus on ranking items based on their discriminatory power without assuming specific functional forms. The analytical pipeline for the npIRT and the pIRT modeling has been previously validated [14–17] as a sufficient and reliable scaling method [18], which offers a promising approach to measuring both SEL and the difficulty of KPs.

In addition to investigating the difficulty of KPs in alignment with diverse student abilities, the main purpose of educational activities is to facilitate the construction of knowledge schemas. The construction process of knowledge schemas involves connecting new KPs with existing knowledge [19]. To effectively assimilate new knowledge, one prerequisite is the acquisition of enough foundational knowledge [20]. During the dynamic process of expanding and shaping knowledge schemas, certain KPs play a pivotal role by introducing other KPs connected to the overarching schema, which are referred to as necessary points [21]. Accordingly, KPs should be sorted sequentially to determine the priority of teaching content. The utilization of a knowledge graph (KG) [22] provides an opportunity for representing KPs as nodes and their relationships as connections. The knowledge graph model (KGM) is the corresponding technical approach to exploring knowledge schemas, enabling the quantitative calculation of the weight of KPs [23].

This study attempts to offer innovative teaching application methods and explore research directions by incorporating student self-evaluation difficulties of KPs, along with IRT and KGM techniques. Pinpointing the difficult points by the IRT addresses the concern of “which KPs demand additional teaching resources for enhanced comprehension” [5]. Determining the important points in the KG tackles the query of “which knowledge points are the indispensable core of this course” [2]. Uncovering the necessary points by the KGM resolves the issue of “which KPs need to be taught first” [24]. The ultimate goal is to customize teaching plans based on the implication of the difficulty and importance of KPs.

## Methods

### Data collection

This study was approved by the Committee for Ethics in Human Research at the Nanjing University of Chinese Medicine (NJUCM), with the issued number as No. 2021-LL-13(L).

A collaborative process involved a three-person voting method facilitated by three rehabilitation professors. These three rehabilitation professors jointly assessed and selected 100 KPs from the curriculum. Following the completion of the final exam, physical therapy students were engaged in the online questionnaire regarding the difficulty rating of KPs. This survey assessed the perceived difficulty of the 100 KPs based on a five-point Likert scale, where students indicated their perception on a scale ranging from 0 (very easy) to 4 (very difficult).

### Statistical tools and methods

**Fig. 1:**
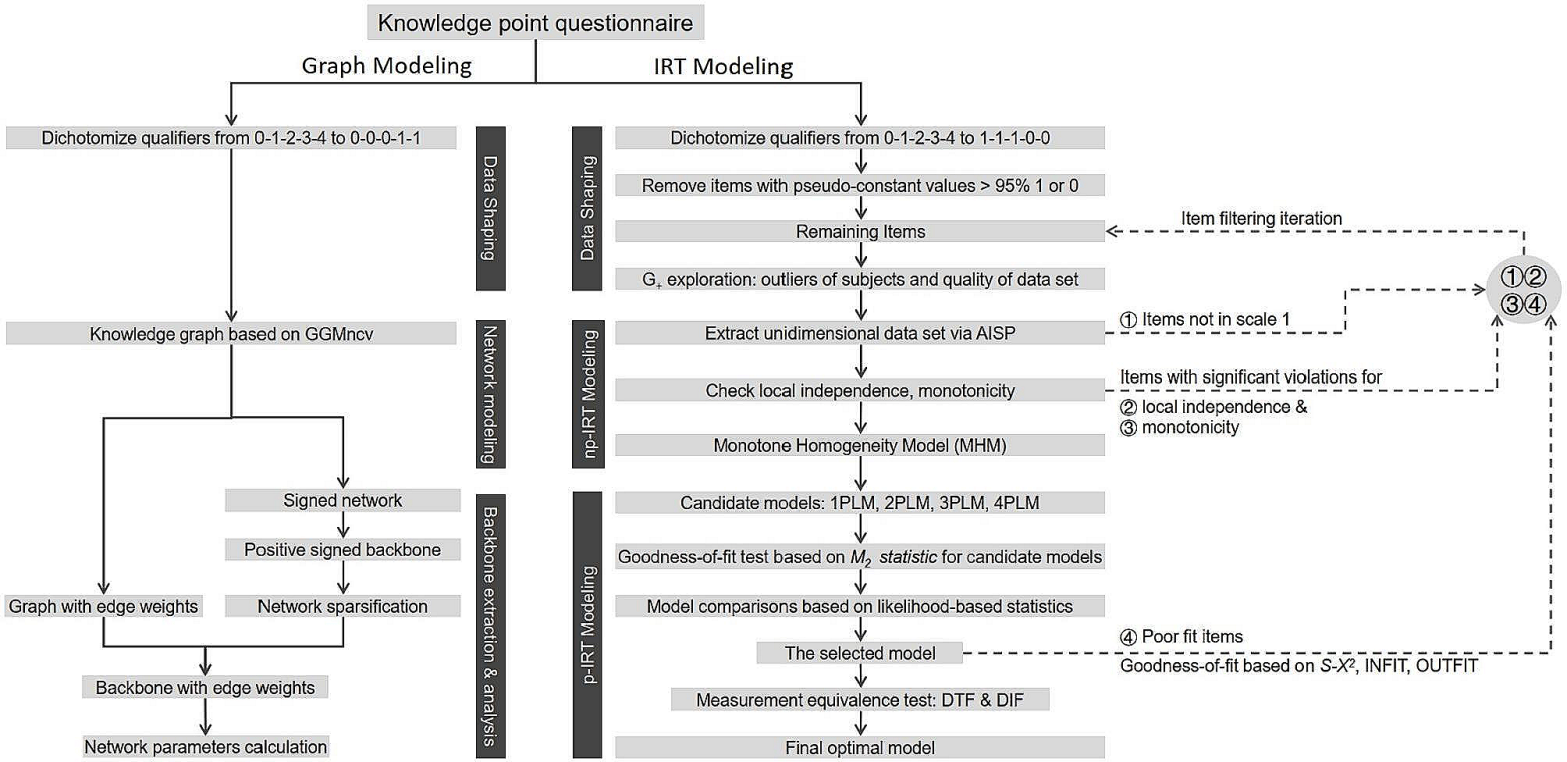
Data processing pipeline AISP: automatic item selection procedure; IRT: item response theory; np-IRT: non-parametric IRT; p-IRT: parametric IRT; 1PLM, 2PLM, 3PLM, and 4PLM: logistic item response model with 1 parameter, and 2, 3, and 4 parameters; INFIT: inlier-sensitive fit/information-weighted fit; OUTFIT: outlier-sensitive fit; S-X2: signed chi-squared test statistic; DTF: differential test functioning; DIF: differential item functioning

The data analysis process, as depicted in Fig. 1, involved several R packages within the R software (Version 4.2.0) [25] to facilitate key steps. We used the *mokken* [26] and *mirt* [27] packages [25] to construct the IRM and parameter estimation. The *ggstatsplot* package [28] was operated for correlation analysis and visualization. The *robustbase* package [29] was employed to analyze the upper and lower bounds of skewed distributions. The *GGMncv* package [30] was conducted for network modeling, while the *backbone* package [31] interpreted the network skeleton structure. The *igraph* package [32] was exploited for network visualization and parameter analysis.

### IRT modelling

#### Data transformation

The difficulty rating scores for KPs were binarized from 0-1-2-3-4 to 1-1-1-0-0. Our study employed the “ascending assignment principle” or assigning by confidence. Under this scoring system, 0 indicated students had self-perceived difficulty mastering certain KPs, whereas 1 indicated students were confident that the knowledge point was easy to learn. A higher questionnaire score (total score of all items) corresponded to greater SEL.

To avoid ceiling and floor effects, the mastery ratio (proportion of KPs with a binary value of 1) and the unfamiliarity ratio (proportion of KPs with a binary value of 0) were calculated for each knowledge point. If KPs with a mastery rate or unfamiliarity rate greater than 95%, they are considered pseudo-constant.

Guttman errors were calculated after removing pseudo-constant KPs. The upper fence of the Guttman error distribution was calculated using the corrected box plot [29]. If Guttman errors exceed the upper fence, it is considered as extreme response bias and would be eliminated. The IRT modeling analysis was conducted as follows. The SEL of students estimated by IRM was defined by the “person ability”, or “θ” value of latent trait. Item difficulty computed by IRM was expressed as the same logit as the person ability. The “outcome” or “learning capacity” implied the academic level measured by exam scores.

#### Mokken scale analysis

Mokken Scale Analysis (MSA) is a npIRT model, which can extract a parsimonious subset of items from the original questionnaire items. The total score of one or more subsets of items informs the ordering of the latent traits. We adopted the monotone homogeneity model (MHM) as one of Mokken models, which relies on three assumptions to order persons using the sum score on a set of items [18]: ① Unidimensionality: The scale measures the single latent trait, equivalent to one factor in the scale; ② Local independence: The associations between scores of two items are solely explained by the θ, where the individual item score is conditionally independent given the latent trait; ③ Monotonicity: Monotonicity is depicted as the item characteristic curve (ICC) that increases or remains constant, but cannot decrease as the θ increases. The ICC is plotted to speculate the relation between the θ and the probability of obtaining item scores, which is typically an S-shaped curve.

The homogeneity coefficients, also known as scalability coefficients, are key indicators of MSA. Considering the sample size and number of questionnaire items, the threshold for the global homogeneity coefficient of all items (denoted as H) was set at 0.42 [33]. According to this boundary value, the automatic item selection procedure (AISP) was exerted to obtain a set of items that meet the unidimensionality [34]. The inter-item homogeneity coefficient (H_ij_) was then calculated, where H_ij_ < 0 violates the MHM assumption.

The conditional association proposed by Straat et al. [35] was also utilized to compute three W indices to identify the local dependence. The W_1_ index detects the positive local dependence (cov(i, j|θ) > 0). The W_2_ index determines the likelihood of each item being in a positive local dependence. The W_3_ index explores negative local dependence (cov(i, j|θ) < 0). The upper limit of the Tukey threshold regarding each W index distribution is the criteria to screen the extreme W values. If W values are larger than the upper limit, it means the violation of local independence. Additionally, we employed the ICC visualization analysis and counted the number of violations to test for monotonicity in MHM.

#### Logistic model analysis

Although the MSA can extract a set of items that meets three assumptions of the MHM, it employs face values rather than parameters to characterize person abilities and item difficulties. On the other hand, the stricter pIRT models have been designed to compare individual abilities and item difficulties on the same scale, which also needs to satisfy unidimensionality, local independence, and monotonicity assumptions. Thus, constructing the MHM and extracting candidate items derived from the MSA are more effective in conducting the pIRT modeling [15, 16, 18, 35–37].

One common unidimensional pIRT model is the logistic item response model. These models implement log odds (Logit) as the unit of measurement for person abilities (θ, i.e., latent traits) and item parameters. Within the logistic model, four key item parameters are illustrated in the fICC [38]: ① Discrimination (a): This parameter corresponds to the maximum slope value at the inflection point on the ICC. It quantifies how effectively items can differentiate between individuals with high and low abilities. ② Difficulty (b): The θ value corresponds to the inflection point on the ICC. As the b value increases, the ICC shifts to the right, indicating an increase in item difficulties, resulting in a decreased scoring rate for test items, even when person abilities remain unchanged. Conversely, a decrease in the b value shifts the ICC to the left, signifying a decrease in item difficulties. ③ Guessing (g): The lower asymptote of the ICC. If g is greater than zero, it indicates that individuals with low ability have a certain probability of obtaining scores due to guessing. ④ Carelessness (u): The upper asymptote of the ICC. If u is less than 1, it suggests that individuals with high ability may lose points due to carelessness.

These parameters are instrumental in constructing four different logistic models. The 1-parameter logistic model (1PLM) estimates the b value, assuming default values of a = 1 (consistent discrimination for all items), g = 0 (no guessing), and u = 1 (no carelessness). The 2-parameter logistic model (2PLM) estimates a and b, with default values of g = 0 and u = 1. The 3-parameter logistic model (3PLM) estimates a, b, and g, while assuming a default value of u = 1. The 4-parameter logistic model (4PLM) estimates all four parameters. The estimations for the four alternative models are conventionally set in the range of -6 to 6 Logit for θ values, and the parameter estimation usually adopts the expectation-maximization (EM) algorithm. The calculation precision (i.e., EM convergence threshold) default is set as 10^− 5^. The assessment of these models was carried out by two-step tests.

First step assessed the goodness of fit (GOF) of the model and the questionnaire data, including *p*-value based upon M_2_ statistic, root mean square error approximation (RMSEA), Tucker-Lewis index (TLI), and comparative fit index (CFI). This study determines the model fit following the criteria: *p* > 0.05 [39], RMSEA < 0.05 [40], TLI > 0.95, and CFI > 0.95 [41].

Second, when multiple models display good fitness, it’s essential to conduct pairwise comparisons using likelihood ratio tests. If the *p*-value < 0.05 signifies a significant difference between the two models, the model with lower Akaike information criterion (AIC) and Bayesian information criterion (BIC) values is preferred. The *p*-value is great than 0.05, which indicates no significant difference between the models. Even though the model with a smaller AIC and BIC might be a reasonable choice in this scenario, it’s important to contemplate the inclusion of g and u parameters. A significant positive correlation between total scores and Gutmann errors indicates that individuals with higher scores tend to make more Guttman errors, likely due to carelessness. In this case, including the u parameter is recommended, leaning towards the 4PLM. Conversely, if there’s a significant negative correlation, it suggests that individuals with lower scores are prone to more Guttman errors, possibly resulting from excessive guessing. Here, the g parameter should be integrated, pointing to a preference for the 3PLM.

#### Analysis of the final model

Four key indicators were employed to evaluate the final model’s internal consistency, including Cronbach’s alpha, Guttman’s lambda-2, Molenaar-Sijtsma statistic, and latent class reliability coefficient (LCRC). As per van der Ark et al. [42], the LCRC stands out as a superior measure of reliability compared to the other three indicators. A value exceeding 0.9 is deemed indicative of high reliability.

Also, we developed the self-report-based knowledge point learning IRT model. The estimated θ values stand for individuals’ SEL in mastering the course material. The correlation analysis was also performed between the θ values and students’ final exam scores in the “Therapeutic Exercise” course (course learning outcomes), as well as their average GPA (comprehensive learning capacity). The aim was to explore the relationship between SEL and both course learning outcomes and comprehensive learning capacity.

The examination of measurement equivalence, also known as measurement invariance, was conducted to revolve around the principle that individuals with the same θ value exhibit score differences attributable to factors other than θ [43–45]. These extraneous factor-related differences can be classified into two categories: differential item functioning (DIF) at the item level, and differential test functioning (DTF) at the overall test level. The exclusion of DIF and DTF can ensure unbiased assessment results across different populations by eliminating potential biases introduced by specific items or scales. DIF analysis is built on the concept of anchor items, which are items exhibiting no significant between-group differences in their parameters. The DIF analysis comprises two steps, each step involving an internal iterative process [45], that is, exploratory DIF analysis and confirmatory DIF analysis.

The initial step involved a stepwise iterative approach, where the assumption of “all other items as anchors (AOAA)” was applied. Each item was sequentially selected to gauge any discernible between-group differences in its parameters (a, b, g, u) using likelihood ratio tests. Any item with a *p*-value > 0.05 was designated as an anchor item, while items with *p-*values < 0.05 were categorized as suspected DIF items. These suspected DIF items were methodically removed one by one until every item had undergone inspection. This process yielded two lists: one consisting of anchor items and another comprising suspected DIF items.

The second step was a systematic iterative process, where the assumption of “the suspected DIF item as the anchor item” was derived from the previously identified anchor items. Each item with suspected differential item functioning (DIF) was incorporated into the model one at a time. We then conducted a likelihood ratio test to evaluate any between-group differences in the item parameters. If an item exhibited a *p*-value < 0.05 and a substantial effect size, it was categorized as a DIF item. Items with *p*-values < 0.05 but with a small effect size, in accordance with the criteria outlined by Meade [44], as well as items with *p*-values exceeding 0.05, were classified as non-DIF items. Upon completing this second-step iteration, the non-DIF items identified during this phase, along with the anchor items from the initial step, were merged to create a conclusive list of non-DIF items. To visualize the results of the second-step analysis, an expected score distribution plot was presented.

In this study, the focus group was the male group, with the female group serving as the reference group. Using the θ values of the focus group as the reference point, we employed the item parameters specific to each group to compute the expected item scores and overall test scores. These calculations enabled the creation of an expected score distribution plot, revealing the comparative performance of both groups.

### KG modeling

#### Data shaping

The knowledge point rating scores were transformed from 0-1-2-3-4 to 0-0-0-1-1. A dichotomization strategy was employed, assigning a score of 1 to KPs categorized as “difficult” or “very difficult”, and a score of 0 to those deemed “easy”, “relatively easy”, or “slightly difficult”.

#### Network preparation

Gaussian Graphical Models with Non-Convex Penalties (GGMncv) were used to compute the partial correlation coefficients between KPs [30]. KPs were considered as network nodes. The partial correlation relationships constituted connections, and the magnitude of the partial correlation coefficients manifested the strength of these connections. This methodology facilitated the construction of a KG rooted in network theory. There was a total of 100 nodes and 1197 connections in our KG. All nodes were interconnected in a singular network structure without any isolated or separate subnetworks.

#### Skeleton extraction

A three-step process was adopted to extract the skeleton structure of the KG [31]. The first step extracted a positive signed backbone through the disparity filter method [46]. The disparity filter determined the significance of the connection values, retaining only those connections that had a significant difference at a significance level of 0.05, with Bonferroni correction for multiple testing. This step led to a 50.7% reduction in connections, transforming the previous network into a signed network, where positive and negative connections were respectively represented as + 1 and − 1.

This yielded a positive signed backbone comprising 104 connections. The positive signed backbone specifically elucidated the positive correlation relationships between KPs, where mastering the knowledge point i aided in comprehending the knowledge point j. 461 negative connections were ruled out due to lack of practical significance, as they meant mastering the knowledge point i would make it more difficult to understand the knowledge point j.

The second step involved network sparsification. The most important connections of each node were extracted from the labeled skeleton with the L-Spar model, as introduced by Satuluri et al. (2011) [47]. The threshold of the L-Spar model was set to 0, which enabled the preservation of the single most crucial connection for each node. This step led to a further reduction of 2.9% in connections. Finally, a sparse positive signed backbone structure emerged, encapsulating 101 connections.

The third step restored the actual connection values. According to the positive signed backbone structure, the corresponding structure containing the actual connection value was extracted from the original network, thus obtaining the positive backbone of the KG. The positive signed backbone structure only included connections with a value of 1, which resulted in a skeleton structure of the positive correlation relationships in the KG. The positive backbone illustrates the most important positive correlation relationship structure in the KG, which could be further utilized to examine the weight of each knowledge point.

#### Network analysis

The term “ego” denoted a specific knowledge point that was selected for examining its weight. ① Degree (DEG) and weighted degree (wDEG): DEG measures the number of connections a given ego node has, while wDEG considers the cumulative strength or weight value of those connections. A higher value indicates that the ego has a greater local impact on the network. ②Betweenness (BET): BET of the ego quantifies the information flow. The range of values is standardized to 0–1. A higher value indicates that the ego serves as a bottleneck, meaning other nodes rely on it to connect. ③ Hub score (HUB): HUB exhibits the centrality of the ego as an information hub within the network. It considers not only the number and strength of connections held by the ego but also the connections of the ego’s neighboring nodes. The range of values is standardized to 0–1, with a higher value signifying a more central position for the ego in the network. ④Laplacian centrality (LAP): LAP measures the extent of disruption to the overall network structure if the ego is removed. The extent of damage will involve the overall network connectivity and structure if the ego is removed. A higher value indicates that the ego is more indispensable to the network.

## Results

### Demographic

218 students (62 male and 156 female) majoring in Rehabilitation Therapy at the NJUCM were enrolled in this study. 10 excluded students took ≤ 100s to complete the questionnaire, and were therefore excluded from this study, as their responses were considered too hasty. The results of the remaining 208 students (56 males and 152 females) were specified for further analysis. The average time to complete the questionnaire was 205.00 s (s) [95% CI: 106.35s, 660.32s]. When stratified by gender, female and male students completed the questionnaire in 212.50s [95% CI: 110.65s, 680.87s] and 189.50s [95% CI: 104.88s, 455.00s] respectively, with no significant gender differences (Kruskal-Wallis χ^2^ = 3.585, df = 1, *p* = 0.0583, *η*^*2*^ = 0.0173). Average final exam scores were 86.00 [95% CI: 63.00, 95.00] for females, and 82.00 [95% CI: 62.38, 95.88] for males, without significant gender differences (Kruskal -Wallis χ^2^ = 3.4216, df = 1, *p* = 0.0644, *η*^*2*^ = 0.0165). The final exam GPA was 3.35 [95% CI: 2.43, 3.98], of which 3.42 [95% CI: 2.46, 3.97] GPA for females and 3.20 [95% CI: 2.41, 4.00] GPA for males. Although there was a significant difference in GPA across gender (Kruskal-Wallis χ^2^ = 9.4402, df = 1, *p* = 0.0021), the effect was small (*η*^*2*^ = 0.0456) and considered negligible.

### IRT modeling results

#### Data preparation

The difficulty rating of 100 KPs was binarized, which did not exhibit constant values. All of them entered the following IRM. There were no pseudo-constant KPs after dichotomization. As also shown in Figure S1, no students were excluded due to exceeding the criterion of the upper limit of Guttman errors, thereby allowing integration of the data collected from 208 students into the subsequent IRM phase.

#### Non-parametric IRT: mokken scale analysis

##### AISP Analysis (Table S1)

The threshold of 0.42 was set for the H, which led to the removal of 33 items that did not align with any specific dimension. There were 67 remaining items divided into 5 dimensions (scales), of which 50 items were in dimension 1. Elevating the H threshold did not increase in the number of items allocated to any dimension. Following the methodology recommended by Straat et al. [33], this study adopted the H threshold of 0.42. Consequently, 50 items from dimension 1 (scale 1) were chosen for further detailed analysis (Table S2).

##### Unidimensionality analysis

The 50-item scale exhibited an H of 0.4756 and a standard error (SE) of 0.0355. In accordance with the criteria established by Sijtsma and van der Ark [26], the homogeneity of our scale was determined to be at a medium level. Given that the extracted items were situated within dimension 1, there was no need for additional assessments of unidimensionality.

##### Local independence analysis

According to Sijtsma and van der Ark [26], if the relationship between any two items i and j violates the local independence given H_ij_ < 0, the MHM was not satisfied. The minimum value of H_ij_ for the 50-item scale was 0.2063, confirming that none of the H_ij_ values violated the prerequisites of the MHM. Moreover, the W_2_ index also affirmed the absence of any items engaged in locally positive dependent relationships [35].

##### Monotonicity analysis

In the monotonicity test, if the diagnostic critical value (Crit) is ≥ 80, it can be considered a significant violation [48]. There were no obvious violations in 50 KPs (Table S3), which conformed to monotonicity. The monotonicity can also be further confirmed by the ICC shape of the model built in the pIRT stage. The monotonicity was achieved when the ICC of each item increased with θ but did not decrease (Figure S2).

#### Parameter IRT: logistic regression model analysis

##### Model-data fit analysis

As shown in Table S4, the *p* values resulted from the M_2_ test for all models yielding a value of 0, but the RMSEA of the 3PLM fell below 0.05. The TLI and CFI for all models exceeded 0.95. Therefore, all models were assigned for pairwise comparisons.

##### Model-model fit comparison

In Table S5, a significant difference (χ^2^_(49)_ = 77.2966, *p* = 0.0061) was observed between the 1PLM and 2PLM (χ^2^_(49)_ = 77.2966, *p* = 0.0061), with 1PLM exhibiting a lower BIC (ΔBIC = -184.2427), suggesting potential superiority to 2PLM. However, there was no significant difference when comparing the 1PLM to either the 3PLM (χ^2^_(99)_ = 88.6090, *p* = 0.7637), or the 4PLM (χ^2^_(149)_ = 121.8965, *p* = 0.9492).

As illustrated in Figure S3, the total scores of the 50-item scale for each student were negatively correlated with the number of Guttman errors (*p* = 1.51 × 10^− 22^). The correlation coefficient (${\widehat{\rho }}_{Spearman}$= -0.61) fell within the range of 0.4–0.7, indicating a moderate correlation according to Akoglu’s standards [49]. This correlation revealed that students with lower scores tended to demonstrate more Guttman errors, implying that they were more likely to guess correctly on items with higher difficulty. This observation underscored the importance of considering guessing behavior in the analysis. Additionally, there was no significant positive correlation, suggesting higher-scoring students did not have more Guttman errors, i.e., they did not lose scores on items with lower difficulty, and thus, the carelessness parameter did not need to be considered.

The 3PLM was ultimately chosen, which did not have a significant difference with 1PLM and additionally incorporated a guessing parameter compared to the 1PLM. Guessing was the lower bound of the ICC, as shown in Figure S2, where a number of KPs (such as kp.75, kp.55, kp.31, and kp.14) had non-zero guessing values. To ensure the property of the 3PLM result, considering the small sample size, we also performed Monte Carlo simulation that generated 500 models with each simulating 1000 response patterns [50].

##### Reliability analysis

Cronbach’s α = 0.9684, Guttman’s λ2 = 0.9689, Molenaar Sijtsma Statistic = 0.9708, LCRC = 0.9776. All four coefficients were > 0.95, indicating good internal consistency for the 50-item scale.

##### Grade-related analysis

**Fig. 2:**
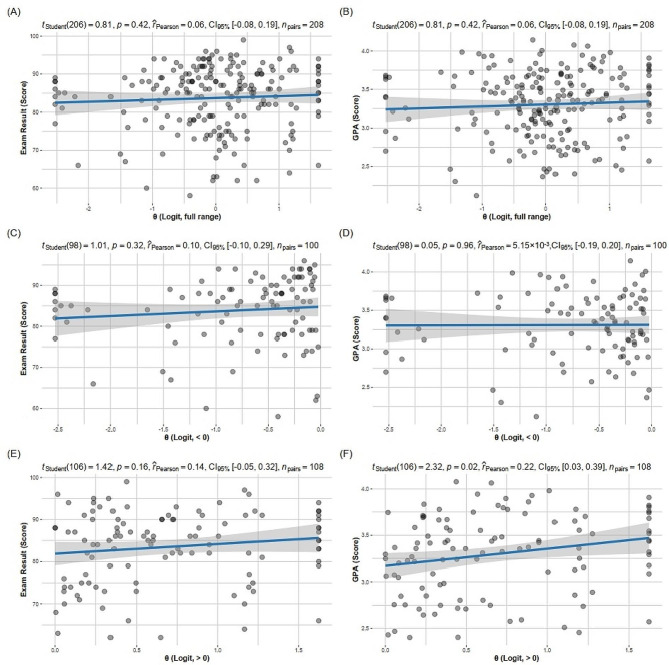
Correlation of exam or GPA scores with full range, < 0, or > 0 values of θ

As shown in Fig. 2, the estimated SEL was not significantly correlated with exam scores (*p* = 0.81) or GPA (*p* = 0.81). However, within the range of θ > 0, a weak positive correlation was observed between GPA and θ (*p* = 0.02, *r* = 0.22).

##### Gender bias analysis

The distribution of expected item scores (Figure S4) and expected test scores (Figure S5) suggested that the 50-item scale did not elucidate significant gender bias.

##### Model parameter analysis

Table 1 displayed the three parameters of the 3PLM arranged in descending order of difficulty. The majority of items illustrated a guessing parameter of either 0 or very close to zero (< 0.1). The item with the highest guessing parameter was kp.31 (g = 0.2803).

Table 1 also displayed the model fit test results for each item. Only kp.14 showed significant differences in the S-X^2^ test (*p* = 0.0466), but its RMSEA (0.0575) reached a publishable level, as suggested by Xia and Yang [41]. Furthermore, the OUTFIT (1.0073) and INFIT (0.9765) for kp.14 both fell within the recommended range of 0.7 to 1.3 according to the thumb rule for item fit [51]. The z-OUTFIT (0.1431) and z-INFIT (-0.131) also fell within the acceptable range of z values (-2.0 to 2.0). Therefore, we concluded that kp.14 fit well in the model.

**Table 1:** Item parameters of the final 3-parameter logistic model composed of 50 knowledge points

Code	b	a	g	S-X^2^	df	p	RMSEA	OUTFIT	INFIT	z-OUTFIT	z-INFIT
kp.35	0.111	1.8069	0	20.5887	18	0.3007	0.0264	0.8292	0.984	-0.8242	-0.206
kp.83	0.1015	1.3937	0.0001	15.4198	20	0.7519	0	0.9099	0.9802	-0.6954	-0.3071
kp.84	-0.0838	1.3301	0	18.567	21	0.6129	0	0.8964	0.9951	-0.9179	-0.0618
kp.33	-0.1	1.6198	0	19.0046	21	0.5848	0	0.8841	0.9856	-0.7362	-0.1933
kp.34	-0.227	1.6857	0	19.2535	20	0.5054	0	0.8726	0.9924	-0.762	-0.0831
kp.41	-0.2901	1.8285	0.0265	21.749	19	0.2969	0.0264	0.9318	0.9857	-0.3659	-0.1628
kp.29	-0.3191	3.069	0	10.6162	15	0.7793	0	0.7009	0.9809	-0.6085	-0.1584
kp.40	-0.3779	2.5496	0	25.4826	17	0.0844	0.0491	0.7345	1.0002	-0.6318	0.0338
kp.28	-0.3956	2.1878	0.0001	16.1194	18	0.5842	0	0.8337	0.9897	-0.5507	-0.087
kp.30	-0.4126	3.4011	0.0407	12.3199	14	0.5806	0	0.7875	0.9698	-0.4125	-0.2282
kp.38	-0.4225	1.9599	0.0001	25.0751	18	0.1229	0.0436	0.8643	0.987	-0.5628	-0.1252
kp.32	-0.4257	1.8216	0	16.0715	19	0.6525	0	0.8936	0.9909	-0.4985	-0.0838
kp.19	-0.4426	1.9338	0	18.1734	18	0.4443	0.0068	0.873	0.9983	-0.5301	0.0082
kp.96	-0.473	1.9743	0.0538	15.7226	19	0.6757	0	0.9971	0.9757	0.0578	-0.2436
kp.55	-0.4826	2.6345	**0.1131**	18.3728	18	0.4314	0.01	0.9547	0.9829	0.019	-0.1294
kp.31	-0.5492	4.8286	**0.2803**	10.9127	11	0.4506	0	0.5692	0.9947	-0.9837	-0.0017
kp.37	-0.5641	3.4274	0	7.8949	13	0.8504	0	0.7525	0.9803	-0.4016	-0.1069
kp.69	-0.6021	2.3274	0.0509	14.024	18	0.7275	0	0.8362	1.0016	-0.398	0.0507
kp.75	-0.6073	3.2072	**0.1331**	18.2176	14	0.197	0.0381	0.8616	0.9847	-0.1762	-0.0784
kp.85	-0.6151	1.6707	0	22.7592	20	0.3008	0.0258	0.9531	0.9851	-0.1918	-0.1403
kp.36	-0.6158	2.4877	0	16.1047	16	0.4457	0.0056	0.7531	1.0096	-0.5014	0.1225
kp.62	-0.6288	3.6468	0	12.8067	9	0.1715	0.0452	0.7694	0.9592	-0.3556	-0.2367
kp.81	-0.6844	2.7747	0	17.9287	15	0.2664	0.0307	0.8223	1.003	-0.2599	0.0664
kp.76	-0.6924	2.7051	0.0001	8.9514	15	0.88	0	0.7284	1.0078	-0.5169	0.1039
kp.39	-0.7082	2.3271	0	19.6627	16	0.2358	0.0333	0.9386	0.9816	-0.0302	-0.1202
kp.64	-0.7087	3.0493	0	17.9881	12	0.1161	0.0491	0.9018	0.9475	-0.025	-0.3394
kp.67	-0.7099	2.565	0.0001	10.9453	15	0.7565	0	0.7289	1.0158	-0.5271	0.1684
kp.47	-0.7148	2.8187	0	12.3859	14	0.5753	0	0.7673	0.9964	-0.3828	0.0166
kp.22	-0.7277	2.4389	0.0001	14.7902	16	0.5401	0	0.7839	1.0095	-0.3749	0.1179
kp.42	-0.7337	2.5247	0	24.1473	15	0.0626	0.0543	0.6919	1.0254	-0.6327	0.2429
kp.73	-0.7469	2.5851	0.0969	7.1165	14	0.9301	0	0.8685	0.9913	-0.1829	-0.0248
kp.57	-0.7474	2.5585	0	12.3626	15	0.6514	0	0.9965	0.9731	0.1399	-0.1703
kp.44	-0.7568	2.6254	0.0001	15.4466	14	0.3483	0.0223	0.7097	1.0169	-0.5758	0.1721
kp.94	-0.7758	1.9691	0	20.52	19	0.3639	0.0197	0.9156	1.0104	-0.2067	0.1311
kp.93	-0.7759	1.9686	0	13.8163	19	0.7943	0	0.9213	1.0099	-0.186	0.126
kp.60	-0.7789	2.3522	0.0001	14.6627	17	0.6198	0	1.1633	0.9679	0.5041	-0.2207
kp.80	-0.8065	2.0092	0	8.1965	19	0.9846	0	0.8301	1.0053	-0.4836	0.0848
kp.49	-0.809	2.2916	0	16.3821	17	0.4969	0	0.7822	1.0112	-0.4285	0.1307
kp.43	-0.8217	2.3311	0	16.3435	16	0.4293	0.0102	0.7452	1.018	-0.4909	0.1826
kp.52	-0.8292	2.8101	0	8.0262	11	0.711	0	0.781	0.9942	-0.3175	0.008
**kp.46**	**-0.8479**	**2.5353**	0.0001	17.7712	13	0.1664	0.0421	0.6533	1.0389	-0.7361	0.3248
kp.51	-0.8541	2.4966	0.0001	13.3625	13	0.4202	0.0116	0.6227	1.0618	-0.8341	0.488
kp.54	-0.8916	2.5223	0.0001	12.7096	13	0.4705	0	0.7054	1.0227	-0.5755	0.2063
kp.50	-0.9043	2.716	0	15.178	12	0.2318	0.0358	0.7702	1.0023	-0.3435	0.0657
kp.21	-0.9088	2.5939	0.0398	15.7297	12	0.2039	0.0387	0.7631	1.0078	-0.405	0.1023
kp.79	-0.9912	2.1361	0	13.4737	13	0.4119	0.0133	0.7702	1.0348	-0.4497	0.2965
kp.14	-1.0289	1.9427	**0.1079**	25.26	15	**0.0466**	0.0575	1.0073	0.9765	0.1431	-0.131
kp.78	-1.0299	2.51	0	12.7831	12	0.385	0.0178	0.786	1.0021	-0.3136	0.0657
kp.24	-1.1351	2.2561	0	13.2	12	0.3547	0.022	0.9918	1.0097	0.1376	0.1137
kp.3	-1.1868	1.8175	0	19.6141	15	0.1872	0.0385	1.2681	0.9516	0.8179	-0.2999

b: item difficulty in Logit; a: item discrimination; g: pseudo-guessing; *S-X*^*2*^: signed chi-squared test statistic; df: degree of freedom; RMSEA: root mean square error approximation of *S-X*^*2*^ test; *p*: *p* value of *S-X*^*2*^ test; OUTFIT: outlier sensitive fit statistic; INFIT: inlier sensitive fit statistic; z-OUTFIT: standardized outfit statistic; z-INFIT: standardized infit statistic

##### Total score conversion

There was a significant positive correlation between the total score (TTS) on the 50-item scale and the model-estimated θ value (*p* = 1.96 × 10^− 235^, ${\widehat{\rho }}_{Spearman}$= 1.0) (Figure S6). The binomial function to facilitate conversion between these two variables can be applied as follows: $\widehat{\theta }= - 2.09+0.0322\times TTS+0.000622\times TT{S}^{2}$.

##### Wright map analysis

**Fig. 3:**
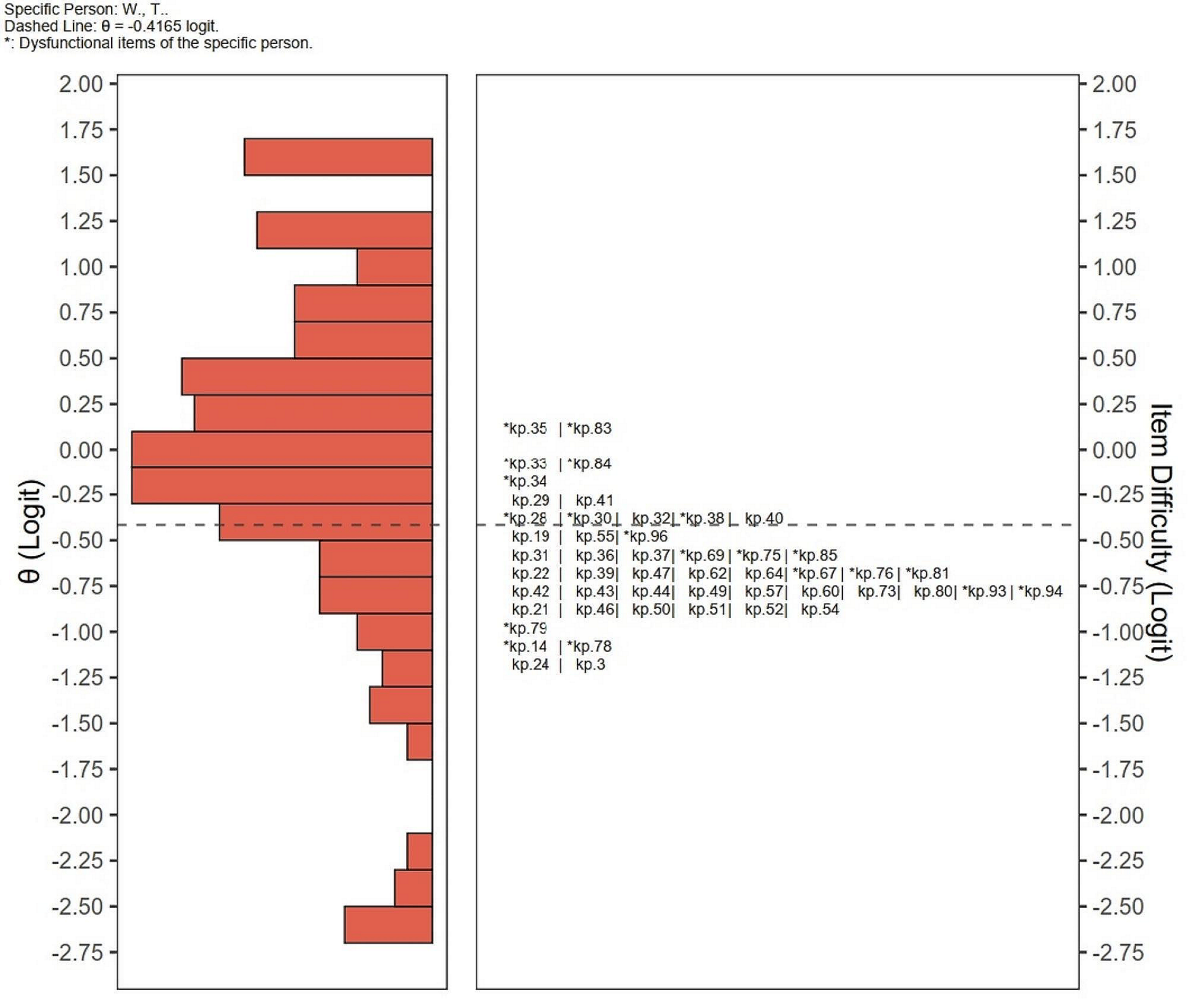
Wright map denoting self-efficacy of student W.,T. The * symbol indicated W.,T received 0 on items in the difficulty rating questionnaire after binarization, which are the items the student perceived as difficult. Knowledge points below the ability line are relatively easy to master, while those above the ability line are relatively difficult. Therefore, the area below the ability line represents competency, while the area above represents challenging points

Figure 3 showed the student W.,T.’s SEL (-0.4165 Logit). For the student W.,T., the items with the lowest difficulty within his learning competency area were identified as kp.14 (Muscular endurance) and kp.78 (Different balance forms). The student W.,T. should prioritize to grasp these KPs.

### Knowledge graph analysis

#### Network parameters for knowledge points

We investigated a KGM composed of 100 KPs, revealing a backbone structure with 68 connections. The connection values were indicated by partial correlation coefficients, ranging from a minimum of 0.106 (weak correlation) to a maximum of 0.464 (moderate correlation) [49].

The analysis of network parameters of KPs within the backbone structure (Table S6) identified 39 isolated points with a degree of 0. The top three KPs in terms of hub score were kp.46 (1.00, Indications for joint mobility techniques), kp.13 (0.8480, Muscle strength), and kp.90 (0.8204, Contraindications for PNF technique). These three points also held the top three in terms of the BET.

The top three KPs considering Laplacian centrality were kp.46 (Indications for joint mobility techniques), kp.13 (Muscle strength), and kp.63 (Indications for joint mobilization). These three KPs also ranked among the top three in terms of weighted degrees. Overall, kp.46 not only featured prominently in the IRM, but also occupied the most critical position within the backbone structure, underscoring its significance in the knowledge structure of the course “Therapeutic Exercise”. According to Table 1, kp.46 had a relatively low difficulty (-0.8479), which fell below the mean difficulty of 50 items (-0.6346). Moreover, it exhibited moderate discrimination (2.5353), closely aligning with the mean discrimination of 50 items (2.42046).

#### Visualization of the main component in the backbone structure

**Fig. 4:**
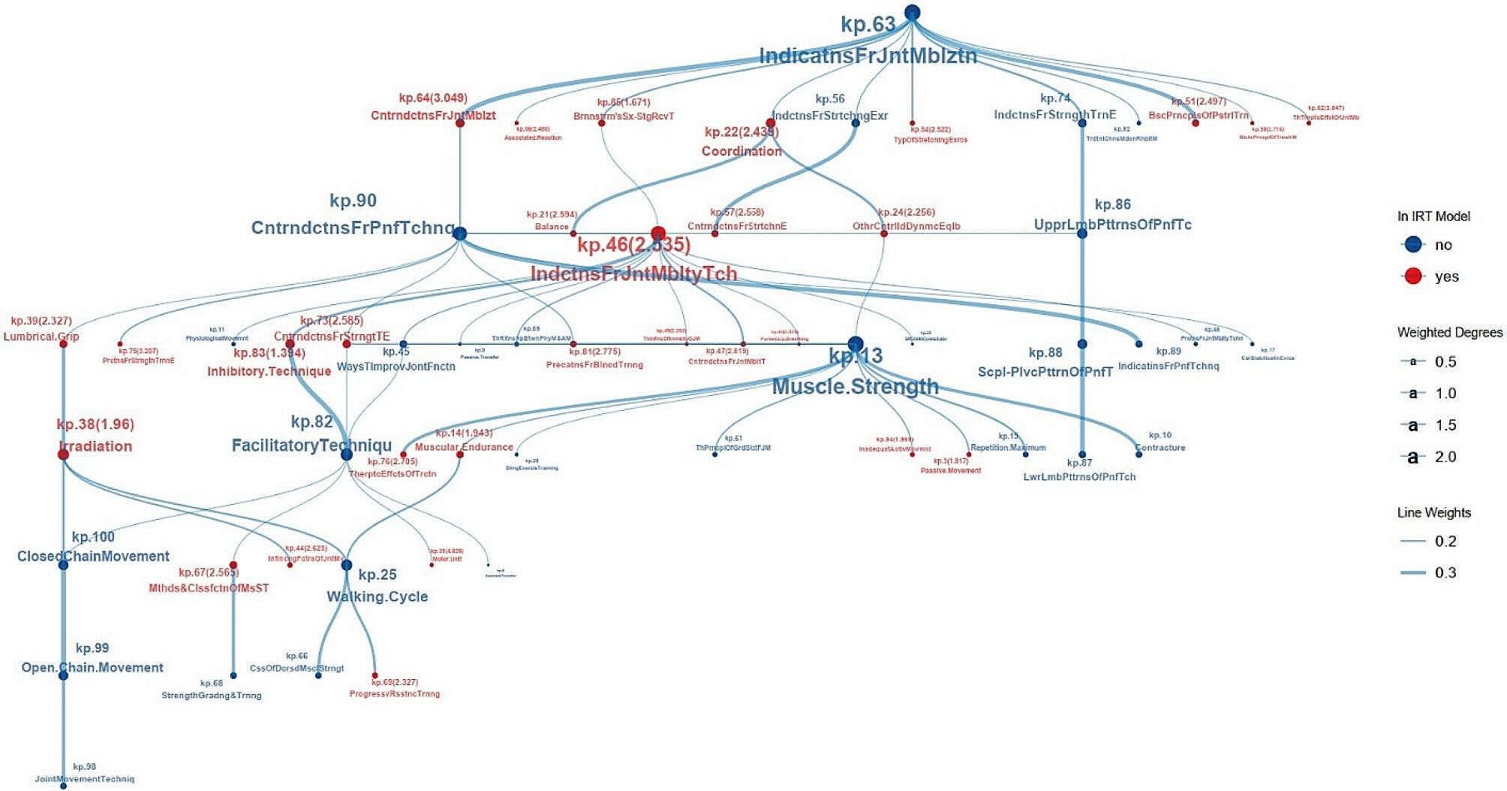
Main component in backbone structure of knowledge graph for Physical Therapy The knowledge points in IRT model (red) were tagged with discrimination parameter

The primary subnetwork within the backbone structure, identified as the main component, comprised 58 nodes, with 29 KPs incorporated into the final IRM. This main component captured 66 out of the 68 connections in the backbone structure. Figure 4 showed the visualization of this main component. The layout was improved by adopting the Sugiyama method for unveiling its hierarchical structure [52, 53].

## Discussion

Adapting teaching strategies stemming from the difficulty of KPs is essential for ensuring quality management in curriculum development. However, there is a lack of current reports that quantitatively assess the self-perceived learning difficulty of KPs in medical education. Course difficulty can be categorized into teaching difficulty and learning difficulty from the perspectives of teachers and students, respectively. To effectively evaluate the learning difficulty of KPs, it is necessary to consider students’ learning capacities.

Clinical education applies a wide range of assessment formats, and structured exams are not consistently employed. This diversity poses a substantial challenge when weighing the difficulty of KPs. Meanwhile, the difficulty of KPs can be intertwined with students’ personal traits. Consequently, integrating comprehensive methods to discern students’ personal traits, evaluate the difficulty of KPs, and understand the correlations between different KPs, is a critical process in achieving pedagogical excellence.

The study extracted 100 KPs from the course “Therapeutic Exercise” to investigate students’ perceived difficulty in comprehending KPs. The npIRT and pIRT modeling were sequentially conducted to obtain a parsimonious item set that could be sufficient to distinguish the personal trait levels of the participants without gender bias. IRM was employed to estimate students’ SEL and item difficulty. Students’ SEL was referred to person ability or θ in the IRM. It should be noted that the interpretation of item difficulties is determined by the binarization strategy, indicating the difficulty of attaining scores. In this study, we assigned value 1 as self-confidence in the questionnaire. Therefore, the practical meaning of item difficulty was the difficulty of being self-confident about mastering certain KPs.

Furthermore, graph modeling techniques were also applied to construct a KG based on the conditional association of difficulty correlations among each knowledge point. Although the KG established in this research might not exactly mirror the knowledge schema formed by students through course learning, it can be used to analyze the knowledge schema affected by personal traits. In other words, it can be regarded as the correlation structure of KPs’ difficulty under the influence of SEL.

### Implication of the IRM-derived student ability

Our result did not find a significant correlation between one-time exam scores and θ values. However, a significant correlation between the GAP and the estimated θ values, particularly within the spectrum of positive θ values. This correlation supports the reliability of the model which contains 50 KPs, in evaluating students’ learning abilities.

Our findings are in line with the research conducted by Richardson et al. [7]. They propose that GPA cannot be solely explained by exam scores, for example, the Scholastic Aptitude Test. They indicate that exam scores are a one-time assessment of course-specific learning effectiveness, while GPA can reveal broader academic performance. GPA is a comprehensive indicator of students’ academic performance, reflecting not only learning abilities but also potential career prospects. They conducted correlation analyses involving GPA during undergraduate university with various traits of students. Their research unveiled a medium-to-large correlation between students’ SEL and GPA, with academic self-efficacy (ASE) exhibiting a medium correlation and performance self-efficacy (PSE) showing a strong correlation. Among the 50 factors they examined, PSE showed the strongest correlation with GPA.

Self-efficacy was first introduced by Bandura to manifest individuals feeling of confidence in their capabilities necessary to reach specific goals [9]. Richardson further defines the self-efficacy into the ASE and PSE. ASE means students’ general perception of their academic competence, which is specifically described as “I have a great deal of control over my academic performance in my courses”. PSE encompasses students’ perceptions of their academic performance capability, as articulated by “What is the highest GPA that you feel completely certain you can attain”. ASE predominantly focuses on self-ability level, while PSE is oriented towards evaluating the anticipated outcomes of the learning process. Our study adopted the difficulty of the KPs scale that was analogous to the concept of PSE as described by Richardson et al. [7], as both aim to gauge the extent of knowledge mastery with complete certainty.

The results of the correlational analysis within this study suggested that SEL, derived from a questionnaire on KPs’ difficulty, can be categorized into two distinct types: positive SEL and negative SEL. For students displaying positive SEL, there was a weak positive correlation between GPA and SEL (*p* = 0.02, *r* = 0.22). It indicated that higher SEL corresponded to higher GPAs, which aligns with self-efficacy theory [9].

On the contrary, this study also identified negative SEL that failed to predict the one-time course exam scores or correlate the comprehensive learning ability measured by GPA. Therefore, it further suggests that SEL based on psychological questionnaires and learning outcomes based on exams should be treated differently. When evaluating students’ learning abilities, reliance on one-time assessment results alone is insufficient.

Furthermore, how to foster positive SEL in students to enhance their overall learning capabilities is also crucial. Encouraging individuals to set realistic and attainable goals can build confidence and contribute to a positive self-perception [54]. The following might offer a practical example to assist students in establishing personal goals regarding their person ability as well as importance and difficulty of KPs.

### Practical example based on the wright map and the knowledge graph

The wright map displays both persons (in terms of their ability) and items (in terms of their difficulty) on the same scale. It was plotted according to individual θ values to assess individual competency, delineating areas of competence (below the θ value) and incompetence (above the θ value). The analysis of a student’s θ was instrumental in pinpointing specific KPs that warrant focused review. Table 1, as provided by 3PLM, offered insights to educators to identify KPs demanding increased attention during future teaching endeavors. KPs characterized by higher difficulty should be allocated more teaching time and resources. Furthermore, KPs with higher discrimination, exemplified by kp.31 (motor unit, with the highest discrimination), in this example, should be subjected to more in-class assessments and feedback. Proficiency in these highly discriminative KPs plays a pivotal role in refining the individual ability.

We selected 100 KPs that were considered important points, while 50-item 3PLM could distinguish the difficulty levels of KPs, so as to figure out the relative difficult points. The excluded items were also important for the course, but they were not “simplified enough to distinguish student abilities in IRT model”. Since the items aside from the model did not have difficulty parameters, how to assess the difficulty of these items was another puzzle that needed to be addressed.

In order to solve the above issue, this study also applied the Gaussian random graph model leveraging conditional correlations to calculate partial correlations between different KPs. The correlation between two KPs within the graph model displayed their “difficulty correlation”. In other words, how likely it was that when one knowledge point was difficult or very difficult, the other knowledge point exhibited a similar difficulty level. Therefore, the KG portrayed the relationships based on difficulty correlations.

An examination of the skeleton structure of the KG, as depicted in Fig. 4, revealed that kp.63 (indications for joint mobilization) occupied the highest position within the hierarchical structure. Notably, it was observed that KPs capable of distinguishing individual traits did not necessarily hold prominent positions within the network. Regarding the principle of constructing graph models based on risk correlation relationships, if important positions are not thoroughly mastered, it would increase the risk of not comprehending other associated items. Therefore, the item kp.46, as a necessary point, which occupied a crucial position and had a certain level of discrimination, should be prioritized for the student to master.

### Limitations and future directions

This study was not without its limitations, which were rooted in the constraints imposed by real-world teaching conditions. These limitations provide opportunities for further improvement.

Firstly, the sample size in our study remained relatively small, and the research was confined to specific courses and KPs. We also observed from the Wright map that a majority of KPs within a similar difficulty range, posing a challenge in distinguishing between individuals with high and low abilities. To enhance the robustness of the findings, we would progressively increase the sample size in each cohort of students in future research. There is also a need to continuously broaden the curriculum by incorporating new KPs and domains, extending the generalizability of our findings. Integrating E-learning platforms with the capability to customize and adapt teaching plans through expert-selected, student-rated questionnaires assessing the difficulty of KPs, holds promise for enhancing the educational experience.

Secondly, our study relied on cross-sectional data, and the difficulty questionnaire was administered only once. While GPAs provide a more comprehensive manifestation of the PSE compared to one-time exams, there is still a requirement for quantitative evidence to support long-term effect of SEL on academic performance. Thus, a promising avenue for future research involves undertaking longitudinal studies to explore the impact of adjusted SEL on long-term academic performance. Our approach could potentially provide a measurement tool for assessing the effectiveness of different interventions aimed at improving SEL over an extended period in future studies.

Thirdly, the validity indicators of the model were singular. Future research should consider supplementing exam scores with other learning ability assessment scales, as well as novel measures like brain-computer interfaces and online learning behavior records. These additions will provide valuable multimodal data to evaluate knowledge point significance and candidate abilities more comprehensively. Despite these limitations, this study introduced an innovative and up-to-date quantitative analysis approach, and its results serve as a foundation for ongoing improvement.

Fourthly, the KGM in this study involved a narrow concept network model which requires to integration of various elements of multiple types such as courses, personnel, and locations, as well as multiple relationship structures. This will enable the incorporation of person abilities and item difficulties calculated by the IRM as indicators for related elements, resulting in a more holistic KG for a comprehensive evaluation of the teaching process [23].

Lastly, the questionnaire was based on students’ self-assessment of the difficulty of KPs, which could reflect the students’ SEL as θ values. Although the IRM defines the θ as personal ability, it might not be directly equated to students’ learning abilities. Nevertheless, the correlation between SEL and GPA provided partial evidence that the questionnaire could also be a useful tool for evaluating learning abilities. Research into the relationships between psychological traits and learning ability traits could become a promising long-term avenue for investigation, and this study contributes practical evidence and tools to this evolving field.

## Conclusion

This study employs a self-assessment questionnaire to achieve students’ perceptions of the difficulty of KPs. It integrates the IRM and KGM to quantitatively assess parameters like students’ SEL, the difficulty level of being self-confident about mastering certain KPs, and importance of KPs. The results affirm that IRM and KGM offer quantitative metrics rooted in empirical data. These metrics are instrumental in identifying and categorizing important, difficult, and necessary points within the curriculum. Furthermore, our study serves as a valuable tool for establishing an evidence-based and refined teaching management approach, thereby enhancing the overall quality of education.

### Electronic supplementary material

Below is the link to the electronic supplementary material.

Supplementary Material 1

## Data Availability

The datasets used and/or analyzed during the current study are available from the corresponding author on reasonable request.
