# Supplementary material for: Interpretation of course conceptual structure and student self-efficacy: an integrated strategy of knowledge graphs with item response modeling
Source: BMC Med Educ. 2024 May 23;24:563. doi: 10.1186/s12909-024-05401-6 (PMC11119392; doi:10.1186/s12909-024-05401-6)
Supplement: Supplementary file 1 — Supplementary Material 1 [file 12909_2024_5401_MOESM1_ESM.pdf]

Supplementary figure

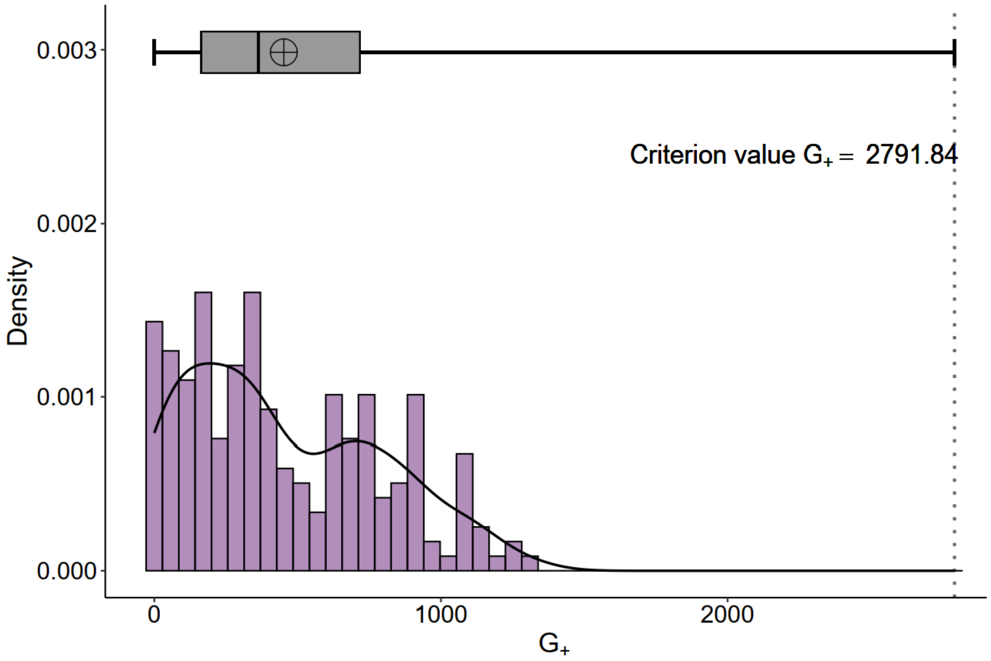

Figure S1 Distribution and adjust boxplot of Guttman errors (G_+)

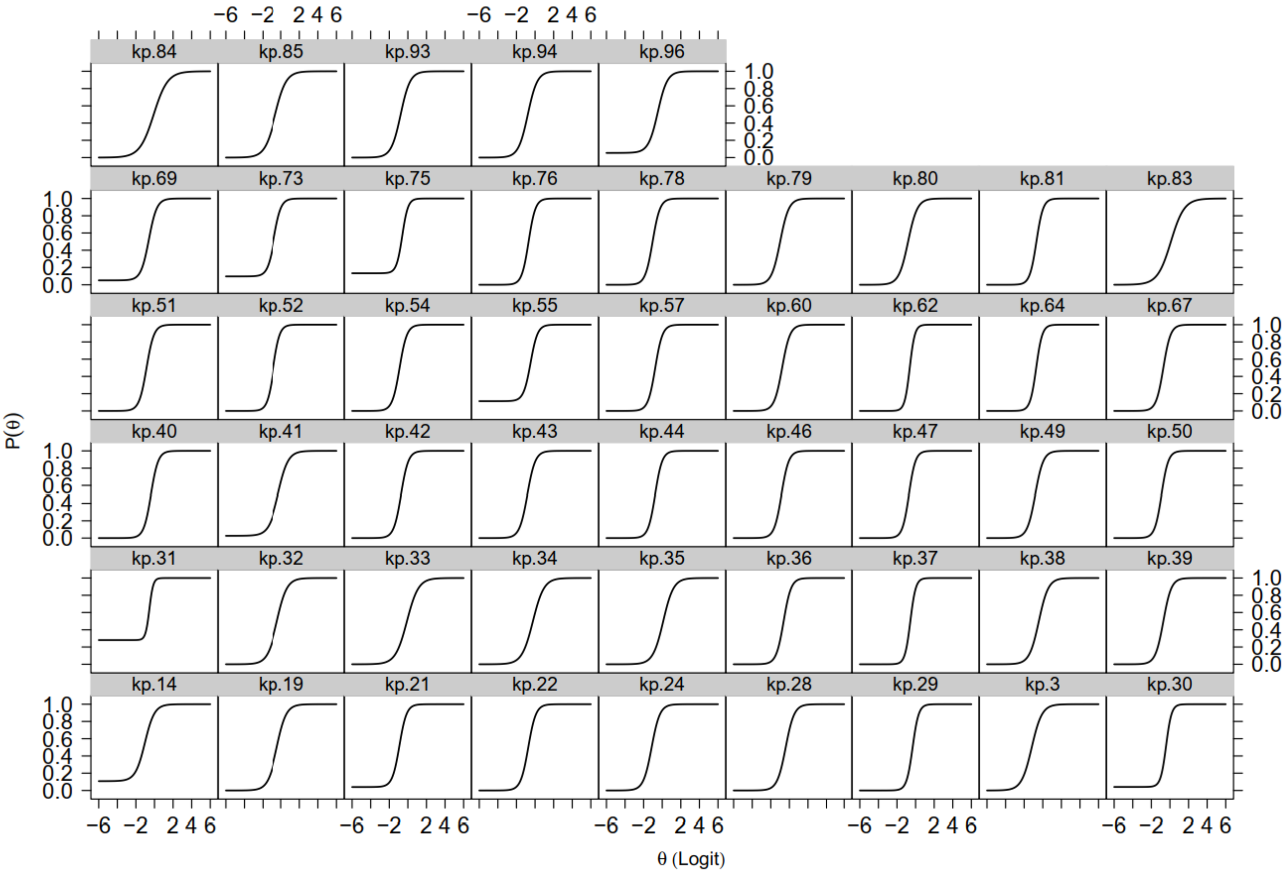

Figure S2 Item characteristic curve for each item in the finally selected model

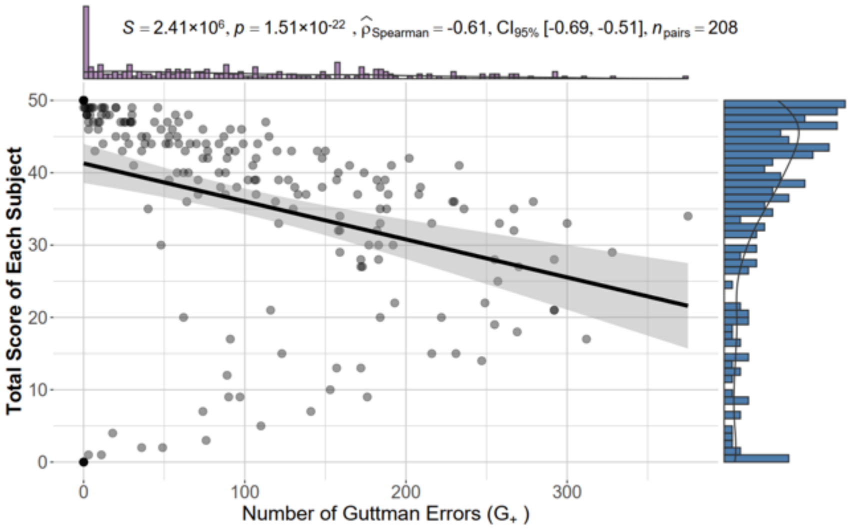

Figure S3 Correlation between total score and number of Guttman errors for 50 knowledge points

S: s-statistic, i.e., sum of all squared rank differences. *p*: *p* value of Spearman rank correlation test. $\hat{\rho}_{Spearman}$: coefficient of Spearman correlation. CI: confidence interval. n_{pairs}: pairs of data for correlation.

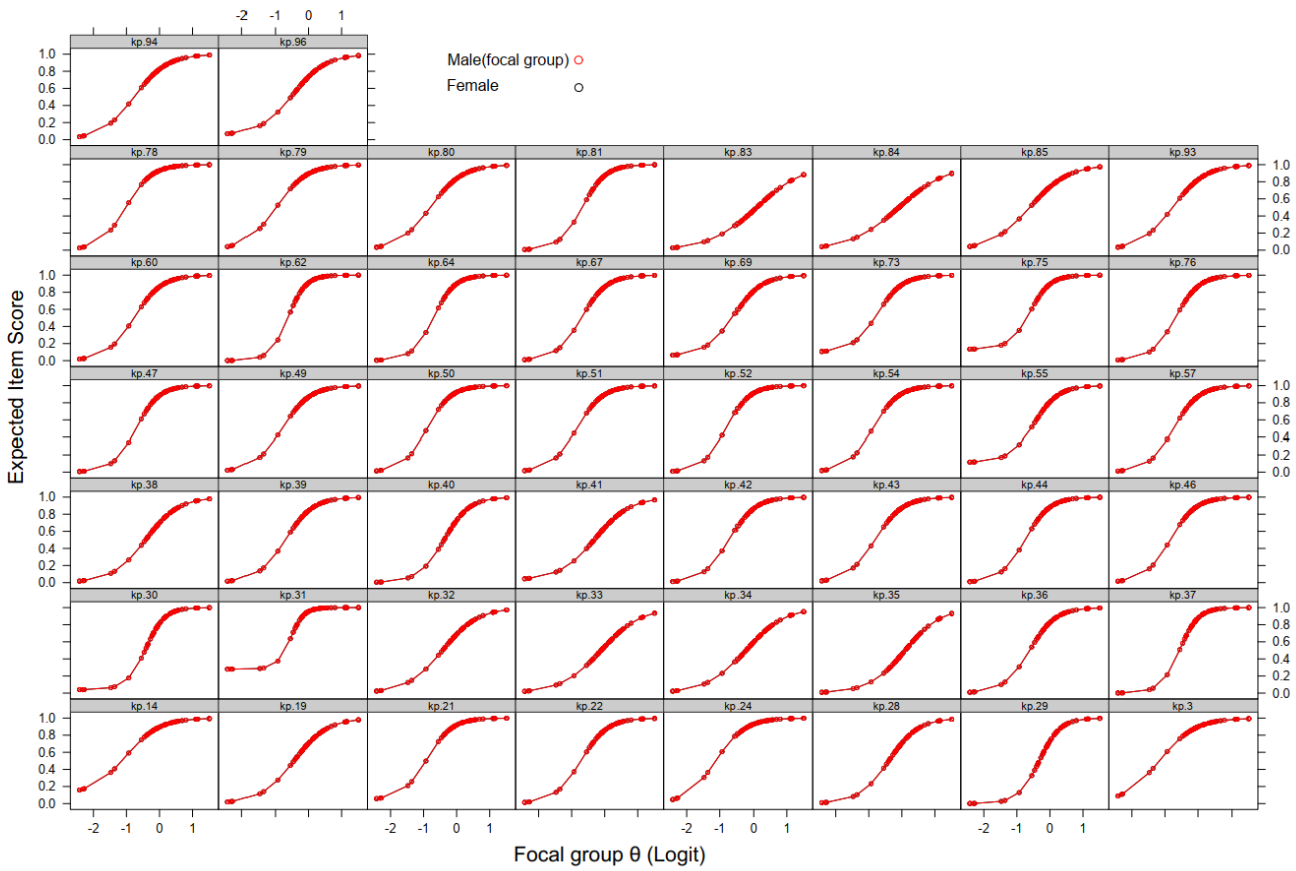

Figure S4 Distribution of expected item scores across the gender groups

50-item scale showed that the score distribution for the focal group (male group, depicted in red) exhibited significant overlap with the reference group (female group, represented in black), resulting in the predominant red coloration.

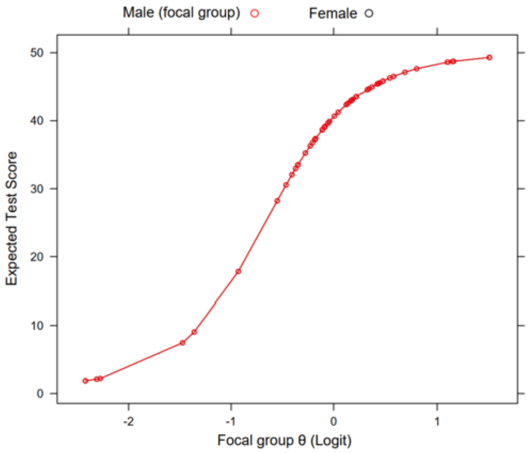

Figure S5 Distribution of expected test scores in across gender groups

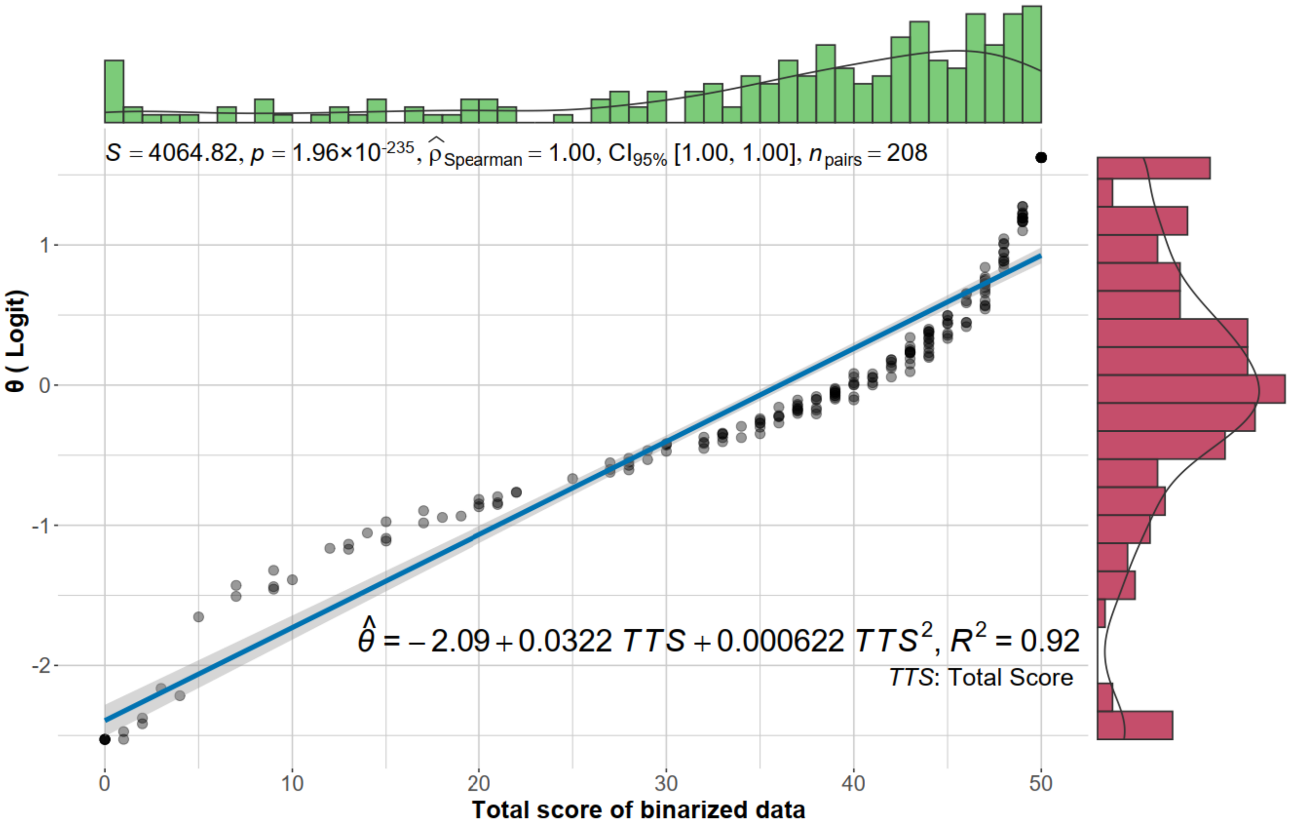

Figure S6 Correlation between total score and θ

Supplementary table

Table S1 Lower bound of Scalability Coefficient H and scale numbers detected via the AISP

Lower Bound	Max scale	Unscalable Items (%)	Items in Scale 1(%)
0.3	8	12	58
0.33	6	10	58
0.36	4	16	66
0.39	5	23	59
**0.42**	**5**	**33**	**50**
0.45	3	44	49
0.48	4	50	38
0.51	3	58	32
0.54	5	62	21
0.57	7	63	12
0.6	5	74	8

Table S2 Knowledge points and their scale when using H=0.42 for AISP

Code	Scale	Knowledge points	Abbreviation	Code	Scale	Knowledge points	Abbreviation
kp.14	1	muscular endurance	Muscular.Endurance	kp.17	2	core stabilization exercise	CorStabilizatinExrcs
kp.19	1	proprioception	Proprioception	kp.23	2	self controlled dynamic equilibrium	SlfCntrlldDynmcEqlbr
kp.21	1	balance	Balance	kp.56	2	indications for stretching exercise	IndctnsFrStrtchngExr
kp.22	1	coordination	Coordination	kp.59	2	The relationship between physiological movement and accessory movement	ThRltnshpBtwnPhyM&AM
kp.24	1	other controlled dynamic equilibrium	OthrCntrlldDynmcEqlb	kp.63	2	indications for joint mobilization	IndicatnsFrJntMblztn
kp.28	1	hemiplegic gait	Hemiplegic.Gait	kp.88	2	Scapula-pelvic pattern of PNF technique	Scpl-PlvcPttrnOfPnfT
kp.29	1	gluteus medius gait	Gluteus.Medius.Gait	kp.99	2	open chain movement	Open.Chain.Movement
kp.3	1	passive movement	Passive.Movement	kp.26	3	drop foot	Drop.Foot
kp.30	1	circle gait	Circle.Gait	kp.61	3	The principle of grade selection for joint mobilization	ThPrncplOfGrdSlctFJM
kp.31	1	motor unit	Motor.Unit	kp.66	3	Causes of decreased muscle strength	CssOfDcrsdMsclStrngt
kp.32	1	key point	Key.Point	kp.70	3	Multi-point isometric training	Multi-PntIsmtrcTrnng
kp.33	1	reflex suppression	Reflex.Suppression	kp.71	3	TENS princeple training	TensPrinceplTraining
kp.34	1	original reflection	Original.Reflection	kp.16	4	resistance training	Resistance.Training
kp.35	1	asymmetrical tonic neck reflex	AsymmetrclTncNckRflx	kp.27	4	knee hyperextension	Knee.Hyperextension
kp.36	1	associated reaction	Associated.Reaction	kp.6	4	postural transfer	Postural.Transfer
kp.37	1	synergy movement	Synergy.Movement	kp.53	5	Affecting Factors of Muscle Strength	AffctngFctrsOfMsclSt
kp.38	1	irradiation	Irradiation	kp.68	5	Strength Grading and Training	StrengthGradng&Trnng
kp.39	1	lumbrical grip	Lumbrical.Grip	kp.1	0	physical therapy	Physical.Therapy
kp.40	1	motor relearning program	MotorRelearningPrgrm	kp.10	0	contracture	Contracture

Code	Scale	Knowledge points	Abbreviation	Code	Scale	Knowledge points	Abbreviation
kp.41	1	exercise prescription	ExercisePrescription	kp.100	0	closed chain movement	ClosedChainMovement
kp.42	1	pursed lip breathing	Pursed.Lip.Breathing	kp.11	0	Physiological movement	Physiological Movemnt
kp.43	1	Aerobic exercise	Aerobic.Exercise	kp.12	0	accessory movement	Accessory.Movement
kp.44	1	Influencing factors of joint movement	InflncngFctrsOfJntMv	kp.13	0	muscle strength	Muscle.Strength
kp.46	1	Indications for joint mobility techniques	IndctnsFrJntMbltyTch	kp.15	0	repetition maximum	Repetition.Maximum
kp.47	1	Contraindications for joint mobility techniques	CntrndctnsFrJntMbltT	kp.18	0	psoas position	Psoas.Position
kp.49	1	The influence of immobility on joint movement	ThInflncOfImmbltyOJM	kp.2	0	therapeutic exercise	Therapeutic.Exercise
kp.50	1	Selection principle of transfer method	SlctnPrncplOfTrnsfrM	kp.20	0	sling exercise training	SlingExercisTraining
kp.51	1	Basic principles of postural transfer	BscPrncplsOfPstrlTrn	kp.25	0	walking cycle	Walking.Cycle
kp.52	1	The effect of stretching exercise	ThEffctOfStrtchngExr	kp.4	0	stretching exercise	Stretching.Exercise
kp.54	1	type of stretching exercise	TypOfStretchingExrcs	kp.45	0	Ways to improve joint function	WaysTImprovJontFnctn
kp.55	1	the procedures of the stretching exercise	ThPrcdrsOfThStrtchnE	kp.48	0	Precautions for joint mobility techniques	PrctnsFrJntMbltyTchn
kp.57	1	Contraindications for stretching exercise	CntrndctnsFrStrtchnE	kp.5	0	continuous passive movement	ContinuousPassvMvmnt
kp.60	1	Grading of joint mobilization procedures	GrdngOfJntMblztnPrcd	kp.58	0	Precautions for stretching exercise	PrctnsFrStrtchngExrc
kp.62	1	The therapeutic effect of joint mobilization	ThThrptcEffctOfJntMb	kp.65	0	Procedure for joint mobilization	ProcedurForJntMblztn
kp.64	1	Contraindications for joint mobilization	CntrndctnsFrJntMblzt	kp.7	0	independent transfer	Independent.Transfer
kp.67	1	Methods and classification of muscle strength training	Mthds&ClssfctnOfMsST	kp.72	0	relationship of Muscle length-tension	RltnshpOfMsclLngth-T
kp.69	1	progressive resistance training	ProgressvRsstncTrnng	kp.74	0	indications for strength training exercise	IndctnsFrStrngthTrnE
kp.73	1	Contraindications for strength training exercise	CntrndctnsFrStrngtTE	kp.77	0	Indications and contraindications for traction	Indctns&CntrndctnsFT

Code	Scale	Knowledge points	Abbreviation	Code	Scale	Knowledge points	Abbreviation
kp.75	1	Precautions for strength training exercise	PrctnsFrStrngthTrnnE	kp.8	0	Assisted transfer	Assisted.Transfer
kp.76	1	Therapeutic effects of traction	TherptcEffctsOfTrctn	kp.82	0	facilitatory technique	FacilitatoryTechniqu
kp.78	1	classification for balance	ClassificationFrBlnc	kp.86	0	Upper Limb Patterns of PNF Technique	UpprLmbPttrnsOfPnfTc
kp.79	1	Affecting Factors of balance	AffectingFctrsOfBlnc	kp.87	0	Lower limb Patterns of PNF Technology	LwrLmbPttrnsOfPnfTch
kp.80	1	training strategy for Balanced training	TrnngStrtgyFrBlncdTr	kp.89	0	indications for PNF technique	IndicatinsFrPnfTchnq
kp.81	1	Precautions for Balanced training	PrecatnsFrBlncdTrnng	kp.9	0	passive transfer	Passive.Transfer
kp.83	1	inhibitory technique	Inhibitory.Technique	kp.90	0	Contraindications for PNF technique	CntrndctnsFrPnfTchnq
kp.84	1	Commonalities of Neurophysiological Therapies	CmmnltsOfNrphyslgclT	kp.91	0	Procedure for exercise prescription	PrcdrFrExrcsPrscrptn
kp.85	1	Brunnstrom's six-stage recovery theory	Brnnstrm'sSx-StgRcvT	kp.92	0	Traditional Chinese Medicine Rehabilitation Methods	TrdtnlChnsMdcnRhbltM
kp.93	1	Inadequate passive movement	InadequatPassivMvmnt	kp.95	0	neurodevelopmental therapy	NeurodevelpmntlThrpy
kp.94	1	Inadequate active movement	InadequatActivMovmnt	kp.97	0	joint mobilization	Joint.Mobilization
kp.96	1	overload	Overload	kp.98	0	joint movement technique	JointMovementTechniq

The items with 0 scale are unscale.

Table S3 The monotonicity test for 50 knowledge points

Code	Knowledge points	Item H	mono_#ac	mono_#vi	mono_#zsig	mono_crit
kp.14	muscular endurance	0.46	3	0	0	0
kp.19	proprioception	0.45	1	0	0	0
kp.21	balance	0.51	1	0	0	0
kp.22	coordination	0.47	1	0	0	0
kp.24	other controlled dynamic equilibrium	0.53	1	0	0	0
kp.28	hemiplegic gait	0.46	1	0	0	0
kp.29	gluteus medius gait	0.53	1	0	0	0
kp.3	passive movement	0.46	1	0	0	0
kp.30	circle gait	0.51	1	0	0	0
kp.31	motor unit	0.51	1	0	0	0
kp.32	key point	0.45	1	0	0	0
kp.33	reflex suppression	0.47	1	0	0	0
kp.34	original reflection	0.46	1	0	0	0
kp.35	asymmetrical tonic neck reflex	0.54	1	0	0	0
kp.36	associated reaction	0.47	1	0	0	0
kp.37	synergy movement	0.53	1	0	0	0
kp.38	irradiation	0.46	1	0	0	0
kp.39	lumbrical grip	0.47	1	0	0	0
kp.40	motor relearning program	0.5	1	0	0	0
kp.41	exercise prescription	0.45	1	0	0	0
kp.42	pursed lip breathing	0.47	1	0	0	0
kp.43	Aerobic exercise	0.46	1	0	0	0
kp.44	Influencing factors of joint movement	0.48	1	0	0	0
kp.46	Indications for joint mobility techniques	0.48	1	0	0	0
kp.47	Contraindications for joint mobility techniques	0.49	1	0	0	0
kp.49	The influence of immobility on joint movement	0.46	1	0	0	0
kp.50	Selection principle of transfer method	0.53	1	0	0	0
kp.51	Basic principles of postural transfer	0.48	1	0	0	0
kp.52	The effect of stretching exercise	0.51	3	0	0	0
kp.54	type of stretching exercise	0.49	1	0	0	0
kp.55	the procedures of the stretching exercise	0.44	3	0	0	0
kp.57	Contraindications for stretching exercise	0.48	3	0	0	0
kp.60	Grading of joint mobilization procedures	0.48	1	0	0	0
kp.62	The therapeutic effect of joint mobilization	0.53	3	0	0	0
kp.64	Contraindications for joint mobilization	0.51	1	0	0	0
kp.67	Methods and classification of muscle strength training	0.47	1	0	0	0
kp.69	progressive resistance training	0.45	3	0	0	0
kp.73	Contraindications for strength training exercise	0.46	1	0	0	0
kp.75	Precautions for strength training exercise	0.47	1	0	0	0
kp.76	Therapeutic effects of traction	0.48	1	0	0	0
kp.78	classification for balance	0.53	1	0	0	0
kp.79	Affecting Factors of balance	0.46	1	0	0	0
kp.80	training strategy for Balanced training	0.43	3	0	0	0
kp.81	Precautions for Balanced training	0.49	1	0	0	0
kp.83	inhibitory technique	0.46	1	0	0	0
kp.84	Commonalities of Neurophysiological Therapies	0.42	1	0	0	0
kp.85	Brunnstrom's six-stage recovery theory	0.42	1	0	0	0
kp.93	Inadequate passive movement	0.43	3	0	0	0
kp.94	Inadequate active movement	0.43	1	0	0	0
kp.96	overload	0.44	1	0	0	0

#ac: count of possible violations in which the item can be involved; #vi: count of actual violations in which the item is involved; #zsig: count significant violations in which the item is involved; Crit: diagnostic critical values.

Table S4 Goodness-of-fit for model and data

M_2	df	p	RMSEA[95%CI]	TLI	CFI	mdl
2245.9248	1224	0	0.0635 [0.0584, 0.0682]	0.9735	0.9736	1PLM
1829.0771	1175	0	0.0519 [0.0462, 0.0571]	0.9824	0.9831	2PLM
1618.535	1125	0	0.046 [0.0398, 0.0517]	0.9861	0.9872	3PLM
1702.7062	1075	0	0.0531 [0.0472, 0.0586]	0.9815	0.9838	4PLM

M_2: a goodness-of-fit statistic with approximate chi-square distribution; df: degree of freedom; *p*: *p* value of M_2 test; RMSEA: root mean square error approximation; CI: confidence interval; TLI: Tucker-Lewis index; CFI: comparative fit index. PLM: logistic item response model with n-parameter(s), n = 1, 2, 3, and 4.

Table S5 Comparison of models through likelihood-based statistics

Model A	Model B	χ^2	p	df	ΔBIC
1PLM	2PLM	77.2966	0.0061	49	-184.2427
1PLM	3PLM	88.6090	0.7637	99	-439.8072
1PLM	4PLM	121.8965	0.9492	149	-673.3966
2PLM	3PLM	11.3124	1.0000	50	-255.5645
2PLM	4PLM	44.5999	1.0000	100	-489.1539
3PLM	4PLM	33.2875	0.9668	50	-233.5894

The comparison of model A with model B with likelihood-based statistics. BIC: Bayesian Information Criterion. ΔBIC: The difference between the BICs of model A and B.

Table S6 Network parameters of knowledge points in backbone structure of knowledge graph model

Code	inIRT	DEG	wDEG	BET	HUB	LAP	Knowledge points
kp.3	yes	1	0.1638	0.0000	0.2081	26	passive movement
kp.14	yes	2	0.3526	0.0187	0.2330	38	muscular endurance
kp.19	yes	0	0.0000	0.0000	0.0000	0	proprioception
kp.21	yes	1	0.2690	0.0000	0.0436	8	balance
kp.22	yes	3	0.6460	0.0343	0.1777	40	coordination
kp.24	yes	2	0.3486	0.0300	0.2517	36	other controlled dynamic equilibrium
kp.28	yes	0	0.0000	0.0000	0.0000	0	hemiplegic gait
kp.29	yes	0	0.0000	0.0000	0.0000	0	gluteus medius gait
kp.30	yes	0	0.0000	0.0000	0.0000	0	circle gait
kp.31	yes	1	0.1323	0.0000	0.1206	16	motor unit
kp.32	yes	0	0.0000	0.0000	0.0000	0	key point
kp.33	yes	0	0.0000	0.0000	0.0000	0	reflex suppression
kp.34	yes	0	0.0000	0.0000	0.0000	0	original reflection
kp.35	yes	0	0.0000	0.0000	0.0000	0	asymmetrical tonic neck reflex
kp.36	yes	1	0.1347	0.0000	0.1052	24	associated reaction
kp.37	yes	0	0.0000	0.0000	0.0000	0	synergy movement
kp.38	yes	4	0.9021	0.0355	0.1299	40	irradiation
kp.39	yes	2	0.4073	0.0283	0.2332	32	lumbrical grip
kp.40	yes	0	0.0000	0.0000	0.0000	0	motor relearning program
kp.41	yes	0	0.0000	0.0000	0.0000	0	exercise prescription
kp.42	yes	1	0.1130	0.0000	0.2454	26	pursed lip breathing
kp.43	yes	0	0.0000	0.0000	0.0000	0	Aerobic exercise
kp.44	yes	1	0.1894	0.0000	0.0319	10	Influencing factors of joint movement
kp.46	yes	12	**1.6916**	**0.1115**	**1.0000**	**204**	Indications for joint mobility techniques
kp.47	yes	1	0.1940	0.0000	0.2454	26	Contraindications for joint mobility techniques
kp.49	yes	1	0.1195	0.0000	0.2454	26	The influence of immobility on joint movement
kp.50	yes	1	0.1273	0.0000	0.1052	24	Selection principle of transfer method
kp.51	yes	1	0.3219	0.0000	0.1052	24	Basic principles of postural transfer
kp.52	yes	0	0.0000	0.0000	0.0000	0	The effect of stretching exercise
kp.54	yes	1	0.1738	0.0000	0.1052	24	type of stretching exercise
kp.55	yes	0	0.0000	0.0000	0.0000	0	the procedures of the stretching exercise
kp.57	yes	1	0.3172	0.0000	0.0275	6	Contraindications for stretching exercise
kp.60	yes	0	0.0000	0.0000	0.0000	0	Grading of joint mobilization procedures
kp.62	yes	1	0.1477	0.0000	0.1052	24	The therapeutic effect of joint mobilization
kp.64	yes	2	0.5289	0.0278	0.3066	46	Contraindications for joint mobilization
kp.67	yes	2	0.3941	0.0115	0.1284	22	Methods and classification of muscle strength training
kp.69	yes	1	0.2308	0.0000	0.0248	10	progressive resistance training

kp.73	yes	3	0.4320	0.0379	0.5301	68	Contraindications for strength training exercise
kp.75	yes	1	0.1671	0.0000	0.2013	20	Precautions for strength training exercise
kp.76	yes	1	0.2606	0.0000	0.2081	26	Therapeutic effects of traction
kp.78	yes	0	0.0000	0.0000	0.0000	0	classification for balance
kp.79	yes	0	0.0000	0.0000	0.0000	0	Affecting Factors of balance
kp.80	yes	0	0.0000	0.0000	0.0000	0	training strategy for Balanced training
kp.81	yes	2	0.2771	0.0096	0.4094	48	Precautions for Balanced training
kp.83	yes	2	0.5923	0.0127	0.3661	44	inhibitory technique
kp.84	yes	0	0.0000	0.0000	0.0000	0	Commonalities of Neurophysiological Therapies
kp.85	yes	2	0.2957	0.0346	0.3507	52	Brunnstrom's six-stage recovery theory
kp.93	yes	0	0.0000	0.0000	0.0000	0	Inadequate passive movement
kp.94	yes	1	0.1562	0.0000	0.2081	26	Inadequate active movement
kp.96	yes	0	0.0000	0.0000	0.0000	0	overload
kp.1	no	1	0.4638	0.0000	0.0000	4	physical therapy
kp.2	no	1	0.4638	0.0000	0.0000	4	therapeutic exercise
kp.4	no	0	0.0000	0.0000	0.0000	0	stretching exercise
kp.5	no	0	0.0000	0.0000	0.0000	0	continuous passive movement
kp.6	no	0	0.0000	0.0000	0.0000	0	postural transfer
kp.7	no	0	0.0000	0.0000	0.0000	0	independent transfer
kp.8	no	1	0.1056	0.0000	0.1206	16	Assisted transfer
kp.9	no	1	0.1144	0.0000	0.2454	26	passive transfer
kp.10	no	1	0.2758	0.0000	0.2081	26	contracture
kp.11	no	1	0.1331	0.0000	0.2454	26	Physiological movement
kp.12	no	0	0.0000	0.0000	0.0000	0	accessory movement
kp.13	no	12	**2.0394**	**0.1069**	**0.8480**	**194**	muscle strength
kp.15	no	1	0.2053	0.0000	0.2081	26	repetition maximum
kp.16	no	0	0.0000	0.0000	0.0000	0	resistance training
kp.17	no	1	0.1259	0.0000	0.2013	20	core stabilization exercise
kp.18	no	0	0.0000	0.0000	0.0000	0	psoas position
kp.20	no	1	0.1215	0.0000	0.2081	26	sling exercise training
kp.23	no	1	0.1066	0.0000	0.2454	26	self controlled dynamic equilibrium
kp.25	no	4	0.8882	0.0282	0.1013	36	walking cycle
kp.26	no	1	0.2909	0.0000	0.0000	4	drop foot
kp.27	no	1	0.2909	0.0000	0.0000	4	knee hyperextension
kp.45	no	3	0.3823	0.0477	0.5742	74	Ways to improve joint function
kp.48	no	1	0.1356	0.0000	0.2454	26	Precautions for joint mobility techniques
kp.53	no	0	0.0000	0.0000	0.0000	0	Affecting Factors of Muscle Strength
kp.56	no	2	0.4632	0.0115	0.1120	30	indications for stretching exercise
kp.58	no	0	0.0000	0.0000	0.0000	0	Precautions for stretching exercise
kp.59	no	1	0.1540	0.0000	0.2454	26	The relationship between physiological movement and accessory movement

kp.61	no	1	0.1687	0.0000	0.2081	26	The principle of grade selection for joint mobilization
kp.63	no	11	**2.0905**	0.0989	0.4288	**166**	indications for joint mobilization
kp.65	no	0	0.0000	0.0000	0.0000	0	Procedure for joint mobilization
kp.66	no	1	0.2547	0.0000	0.0248	10	Causes of decreased muscle strength
kp.68	no	1	0.2749	0.0000	0.0315	6	Strength Grading and Training
kp.70	no	0	0.0000	0.0000	0.0000	0	Multi-point isometric training
kp.71	no	0	0.0000	0.0000	0.0000	0	TENS princeple training
kp.72	no	0	0.0000	0.0000	0.0000	0	relationship of Muscle length-tension
kp.74	no	2	0.5111	0.0073	0.1689	34	indications for strength training exercise
kp.77	no	0	0.0000	0.0000	0.0000	0	Indications and contraindications for traction
kp.82	no	7	1.0906	0.0704	0.4916	86	facilitatory technique
kp.86	no	3	0.7634	0.0292	0.2594	38	Upper Limb Patterns of PNF Technique
kp.87	no	1	0.3505	0.0000	0.0166	6	Lower limb Patterns of PNF Technology
kp.88	no	2	0.6766	0.0115	0.0677	14	Scapula-pelvic pattern of PNF technique
kp.89	no	1	0.3384	0.0000	0.2013	20	indications for PNF technique
kp.90	no	9	1.4742	**0.1076**	**0.8204**	144	Contraindications for PNF technique
kp.91	no	0	0.0000	0.0000	0.0000	0	Procedure for exercise prescription
kp.92	no	1	0.1478	0.0000	0.1052	24	Traditional Chinese Medicine Rehabilitation Methods
kp.95	no	0	0.0000	0.0000	0.0000	0	neurodevelopmental therapy
kp.97	no	0	0.0000	0.0000	0.0000	0	joint mobilization
kp.98	no	1	0.2761	0.0000	0.0104	6	joint movement technique
kp.99	no	2	0.6491	0.0115	0.0426	14	open chain movement
kp.100	no	3	0.7259	0.0300	0.1630	38	closed chain movement

inIRT: whether this item in the final IRT model or not; DEG：degree centrality; wDEG: weighted degree centrality; BET: betweenness centrality; HUB: hub score; LAP: Laplacian centrality.
